# Modeling of Myotonic Dystrophy Cardiac Phenotypes in *Drosophila*

**DOI:** 10.3389/fneur.2018.00473

**Published:** 2018-07-16

**Authors:** Mouli Chakraborty, Beatriz Llamusi, Ruben Artero

**Affiliations:** ^1^Translational Genomics Group, Incliva Health Research Institute, Valencia, Spain; ^2^Interdisciplinary Research Structure for Biotechnology and Biomedicine (ERI BIOTECMED), University of Valencia, Valencia, Spain; ^3^CIPF-INCLIVA Joint Unit, Valencia, Spain

**Keywords:** cardiac dysfunction, myotonic dystrophy, Muscleblind, CTG expansion, CCTG expansion, *Drosophila* disease model, drugs

## Abstract

After respiratory distress, cardiac dysfunction is the second most common cause of fatality associated with the myotonic dystrophy (DM) disease. Despite the prevalance of heart failure in DM, physiopathological studies on heart symptoms have been relatively scarce because few murine models faithfully reproduce the cardiac disease. Consequently, only a small number of candidate compounds have been evaluated in this specific phenotype. To help cover this gap *Drosophila* combines the amenability of its invertebrate genetics with the possibility of quickly acquiring physiological parameters suitable for meaningful comparisons with vertebrate animal models and humans. Here we review available descriptions of cardiac disease in DM type 1 and type 2, and three recent papers reporting the cardiac toxicity of non-coding CUG (DM1) and CCUG (DM2) repeat RNA in flies. Notably, flies expressing CUG or CCUG RNA in their hearts developed strong arrhythmias and had reduced fractional shortening, which correlates with similar phenotypes in DM patients. Overexpression of Muscleblind, which is abnormally sequestered by CUG and CCUG repeat RNA, managed to strongly suppress arrhythmias and fractional shortening, thus demonstrating that Muscleblind depletion causes cardiac phenotypes in flies. Importantly, small molecules pentamidine and daunorubicin were able to rescue cardiac phenotypes by releasing Muscleblind from sequestration. Taken together, fly heart models have the potential to make important contributions to the understanding of the molecular causes of cardiac dysfunction in DM and in the quick assessment of candidate therapeutics.

## Introduction

Myotonic Dystrophy (DM) is characterized by autosomal dominant inheritance and multisystem involvement. Progressive myotonia, muscle degeneration, early onset cataracts, heart defects, neurological problems and endocrine disorders are the most observed multisystemic dysfunctions (1, 2). To date, two distinct forms of DM have been identified. DM1 is caused by an unstable CTG repeat expansion in the 3′UTR of the DMPK gene (OMIM 605377) (3–5). DM2 is caused by an abnormal CCTG expansion in the first intron of the CNBP gene [previously known as zinc finger 9 gene, ZNF9; OMIM 116955] (6–8). Both types share the common disease characteristics, however, they also have distinct clinical features. Prominent distal muscle involvement, marked myotonia and severe congenital form are seen in DM1 whereas DM2 is characterized by prominent proximal muscle involvement, mild myotonia, and absence of congenital form. Clinically, DM2 is milder than DM1 (9). At the molecular level the mutant transcripts [C(C)UG] accumulate in foci leading to disruption of key cellular pathways, namely, RNA processing (10), localization (11), and translation (12, 13). These mutant transcripts alter the muscleblind-like and CUGBP and ETR3 like factor families of RBPs and results in abnormal expression of fetal isoforms of several genes in adult tissues (14, 15). In addition, deregulation of microRNA and RAN-translation may be important additional mechanisms of DM pathophysiology (16–19). Different vertebrate and invertebrate animal models have been successfully generated by different laboratories to understand the disease pathomechanisms. Most of the animal models have been paramount to understand the muscle-related pathomechanisms (20–22). However, till date, only a few reports are available about animal models to study DM heart problems (23, 24). Interestingly, *Drosophila* has been shown to mimic DM cardiac dysfunctions (25, 26). The purpose of this review is to gather all the available information about *Drosophila* cardiac dysfunction models in DM, which are found to complement functional data coming from murine models.

### Heart-related alterations in DM1

Approximately 80% of the DM1 patients will develop the cardiac disease in their lives but the risk is more pronounced in young patients (2–30 years old) than in the old ones (27). Indeed, the cardiac complications account for 30% of patient deaths (28–30). The cardiac involvement mainly includes degeneration of conduction system caused by myocardial fibrosis (31). Myocardial fibrosis is due to myocyte hypertrophy, focal fatty infiltration, and also lymphocytic infiltration (32, 33). This are affecting 40% of the DM1 patients (34) and 65% patients have an abnormal ECG. The typical ECG abnormalities include prolongation of PR interval (>240 ms; 20–40% patients) and the QRS duration (>120 ms; 5–25% DM1 patients) (35).

Conduction disturbances can cause conduction block, ectopic activity, and re-entrant arrhythmias. These disturbances give rise to palpitations, syncope and sudden cardiac death (36). Both atrial and ventricular arrhythmias can occur in DM1 patients. Around 25% of the DM1 patients show atrial (supraventricular) tachyarrhythmias, specifically atrial fibrillation and atrial flutter (30, 34). Ventricular arrhythmias are less common but more severe and are considered as the main cause of sudden death (37, 38).

DM1 patients are also prone to develop structural cardiomyopathy (39, 40). Early in the disease course, the left ventricular diastolic dysfunction is more pronounced than the systolic dysfunction (41). In addition, left atrial dilatation may also occur (33). This impaired relaxation of the cardiac muscle or myocardial myotonia is the cardiac equivalent of skeletal muscle problems in patients (41, 30). Other associated heart manifestations include angina (both stable and unstable), and myocardial infarction. Mitral valve prolapse has been identified in 13–40% of patients and was directly related to stress-induced ejection fraction problem. In some DM1 patients, pulmonary failure was also observed (42).

### Heart-related problems in DM2

Generally, heart dysfunction in DM2 was considered less severe and frequent than in DM1 (43–45). However, recent studies indicate that the total risk of cardiac disease in DM2 is very close to DM1 (9). Like in DM1, DM2 cardiac manifestations include AV blocks, arrhythmias, and dilated cardiomyopathy (46). The subclinical myocardial injury causes conduction defects and is directly correlated with the ECG abnormalities found in DM2 patients (42). Conduction defects also cause severe arrhythmias and sudden death in DM2 patients (40). In contrary to DM1, DM2 patients do not show pulmonary failure (42).

### Murine models to study heart dysfunction in DM

Different mice models have been created to understand the cardiac aspect of the disease. These are, (1) overexpression of expanded (DMSXL) (24) or (2) non-expanded (Tg26) DMPK (47), (3) Cre-lox inducible heart-specific expression of CUG repeats [EpA960] (23), (4) inducible expression of DMPK 3'UTR with short repeats [GFP-DMPK-(CTG)5] (48), (5) compound loss of Mbnl1 and Mbnl2 (49), and (6) CUGBP1 overexpressing mice (50). All of these mice models reproduce DM1-specific cardiac dysfunction to some extent but they do have some specific limitations. EpA960 mice have shown DM1-like ECG recordings, arrhythmia and AV block, but they were so seriously affected that died very early. The DMSXL mice reproduced important clinical aspects as observed in the disease including reduced muscle strength, lower motor performance, and respiratory impairment, but cardiac phenotypes of DMSXL required challenging by the class-I antiarrhythmic agent flecainide (51). In addition, missplicing defects were mild. The GFP-DMPK-(CTG)5 mice showed toxicity within the normal range of repeats in the absence of ribonuclear foci, and had a high rate of mortality. Finally, Mbnl loss of function or CUGBP1 overexpression is not representative of the disease complexity. Therefore, investigation of physiopathological pathways and testing of drugs still needs development of additional whole animal models.

### The *Drosophila* heart as alternative to vertebrate cardiac models

**Figure 1:**
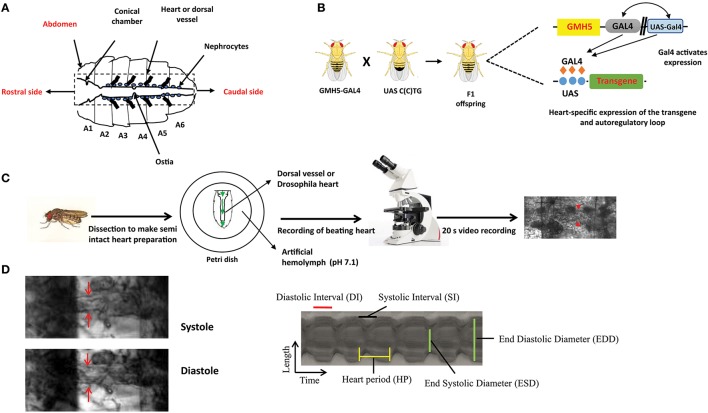
The overall experimental set up for determination of *Drosophila* cardiac parameters. **(A)** Schematic representation of *Drosophila* heart or dorsal vessel. The heart extends from A2 to A6 abdominal segment. The conical chamber, which is present at the beginning of the dorsal vessel. The ostia which are the openings in the heart help to redistribute hemolymph from the heart to the body cavity. The bold lines near the heart represent alary muscle which connect the heart to the cuticle. The pericardial cells or nephrocytes are marked in the figure which has kidney like functions. The rostral and the caudal side of the heart are marked respectively. **(B)** Gal4-UAS system is used to express transgenes in the *Drosophila* heart. GMH5-Gal4 flies are crossed with UAS strains carrying the different length of repeats to drive the repeat expression in the F1 offspring. **(C)** F1 female flies are anesthetized, dissected and maintained in aerated artificial hemolymph solution (pH 7.1). The dissected hearts are recorded for 20 s with a high-speed digital video camera and processed with SOHA. The red arrow represents the *Drosophila* heart. **(D)** A *Drosophila* heart is marked at systole and diastole phase. A representative 2D kymograph indicates heart period (HP), end systolic and end diastolic diameters (ESD and EDD), systolic and diastolic intervals (DI and SI).

The *Drosophila* heart has remarkable similarities with vertebrates in terms of structure and developmental regulation. A common developmental origin has been found on bilaterally symmetrical rows of mesodermal cells which migrate and fuse to form a heart tube at the midline (52). Upon subsequent looping and septa formation, the fly heart is further divided by an intracardiac valve into an aorta and a 1 mm long pulsatile posterior dorsal vessel or proper heart (Figure 1A) (54–56). The *Drosophila* heart also possesses a bilateral pacemaker system. The chief pacemaker situated near aorta expels hemolymph anteriorly whereas, the minor pacemaker placed in conical chamber allows backtracking of hemolymph flow (53, 57, 58). Nevertheless, the mechanism behind the origin of pacemaker potential has not been elucidated (58). So making direct extrapolation of *Drosophila* results to mammals is difficult. In contrast, the fly heart is different in two critical aspects. Calcium channels, instead of sodium channels, are more important to generate heart action potentials in flies. *Drosophila* has a very simple tubular-like heart structure without definitive atria or ventricle structures. However, the implementation of advanced electrophysiological techniques will help to closely describe fly heart functioning and may potentially discover additional levels of fundamental conservation between *Drosophila* and mammals.

The advantages of invertebrate genetics are utilized nowadays to study cardiac development and model diseases. Compared to mice, fly models are easy to create and maintain. In general, high mortality and low breeding rate limit the usage of mice. Chief among *Drosophila* genetic tools, is the ability to target a specific transgene expression to virtually any fly tissue and developmental time in a time frame of 6–8 weeks. This normally requires the binary Gal4-UAS system derived from yeast (59). Gal4 flies control the tissue-specific expression of yeast Gal4 transcription factor through promoters of interest and UAS flies carry specific UAS sequence upstream of the transgene of interest, which is expressed upon crossing with different Gal4-drivers. The effects of tissue-specific gene expression are observed in the progeny (Figure 1B). For example, in F1 offspring the Hand-Gal4 strain drives expression of the UAS-transgene to embryonic cardiogenic mesoderm (60) and tinC-Gal4 drives cardioblast-specific expression of transgenes (61).

## *Drosophila* models of cardiac dysfunction reproduce aspects of DM1 and DM2 pathology

In order to model DM1 and DM2 cardiac dysfunction in flies, UAS-CTG and UAS-CCTG fly lines carrying either 250 CTG or 1100 CCTG non coding pure expansions were generated, respectively, which are within the pathological range reported in the patients (62, 63). As controls, flies carrying 20x repeats were generated. The UAS fly lines were crossed with the cardiac-specific driver GMH5–Gal4 to express the repeats in the heart. The cardiac dysfunction phenotypes of F1 flies expressing repeats in the cardiomyocytes were analyzed at several levels:

### DM1-like molecular alterations

At the molecular level, it has been shown that Muscleblind-like proteins are sequestered in ribonuclear foci and play a prominent role in the disease manifestation. In control *Drosophila*, Muscleblind was not detected in the embryonic heart (64) but in adult cardiomyocytes, it is clearly detected. In the fly heart cells, Muscleblind displayed a dispersed expression throughout the nucleus and cytoplasm (25). Fluorescence *in situ* hybridization (FISH) followed by immunofluorescence technique showed that, upon long CUG or CCUG repeats expression in the cardiomyocytes, Muscleblind became sequestered into ribonuclear foci. In contrast, flies expressing a small number of either type of repeats did not show any foci or Muscleblind accumulation (25, 26).

Muscleblind sequestration leads to missplicing of several important transcripts such as CLCN1, CaV1.1channel, and IR causing different disease phenotypes such as myotonia, muscle weakness, and insulin resistance, respectively (65–67). In DM fly hearts, the inclusion of exon 13 of Serca gene and exon 16 of Fhos gene was significantly altered. These data established that Muscleblind functional depletion observed in DM1 and DM2 fly hearts is due to Muscleblind sequestration in foci (26).

It has been shown previously that expression of the long CTG repeats induces autophagy and has been proposed to cause muscle atrophy in flies (68). Among different autophagy-related genes, expression of Atg4, Atg7, and Atg12 was found to be significantly upregulated in fly muscles expressing the repeats (68). Importantly, these genes were also overexpressed in case of either long CUG or CCUG repeats expression in heart, compared to control flies expressing GFP or short repeats. These data highlighted, for the first time, a potential role for dysregulated autophagy pathway in DM cardiac dysfunction upon expression of expanded repeats (26). Nevertheless, the mechanistic connection between autophagy and heart defects in flies is still missing.

### Cardiac performance of DM flies

**Figure 2:**
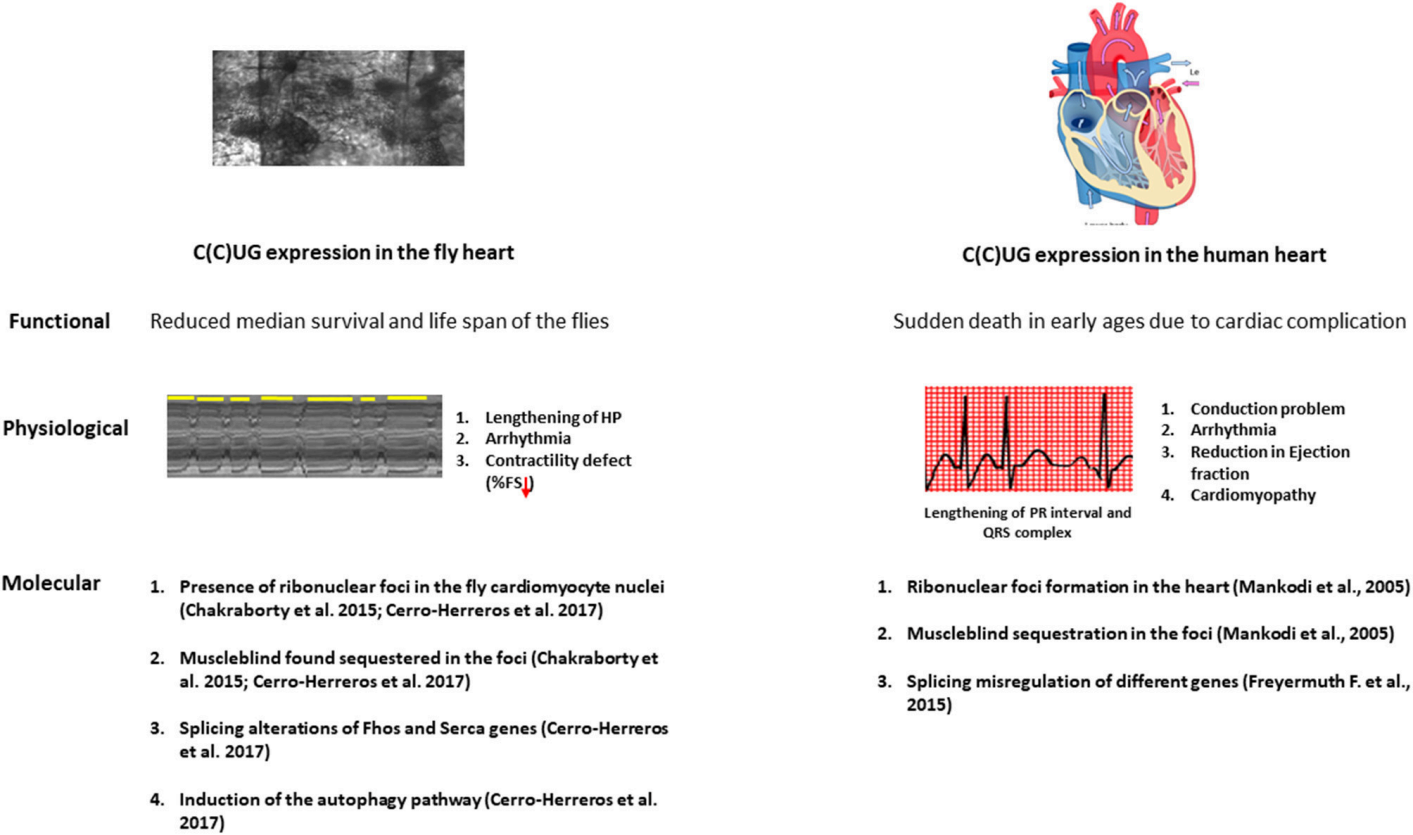
The physiopathological parallelisms between model fly heart and diseased human heart. Repeat expansion in both fly and patient heart causes a marked reduction in the lifespan. The expression of the repeats in the fly heart causes conduction defects, arrhythmia and contractility defects as observed in DM patients. At the molecular level, microsatellite expansion in the heart causes Muscleblind sequestration in the ribonuclear foci and this sequestration leads to misregulation in alternative splicing both in fly and human heart. Induction in the autophagy is also observed in the fly heart.

Mature fly hearts were dissected in artificial hemolymph to record heart-beating with a high-speed video camera in order to study heart function (Figure 1C) (for a detailed description see (69)) (70). Heartbeats are analyzed using SOHA method for quantifying different parameters (71). It generates records of heart wall movement with high-resolution known as M-modes which illustrate the rhythmicity and the dynamics of the heart contractions (Figure 1D) (72). It allows quantification of the following parameters: relaxation and contraction phase (DI, and SI, for diastolic and systolic interval), heart period (HP), arrhythmia index (AI), end systolic diameter (ESD), end diastolic diameter (EDD) and the percentage of fractional shortening (%FS, FS = EDD – ESD/EDD × 100), which is a measure of heart's contractility (Figure 1D). It has been observed that expression of C(C)UG repeats in cardiomyocytes resulted in prolongation of HP. This increasement occurred via increased DI and SI. Reduction in %FS, and increased AI were also seen. SI and DI were more affected in DM2 flies than in DM1 flies (Figure 2). Surprisingly, short repeat expression in heart produced a significant lengthening of systolic interval and this prolongation was Muscleblind independent as foci were absent (26).

### Functional assays

To assess the functional consequences of the expanded repeat expression, survival curves, and climbing, and flying performance tests were obtained from DM model flies. A significant reduction in survival, almost to half as compared to the control flies, was observed upon expression of expanded C(C)UG. Of note, flies expressing short repeats have similar survival to that of control flies. However, climbing velocity and flying performance of these model flies were not affected. These data suggested that reduction in the %FS of these model flies did not affect acute workload demands (flight, and climbing), but did have an important detrimental effect on life-span (26).

## Testing of candidate therapeutics in the *Drosophila* cardiac dysfunction model of DM1

To determine whether DM transgenic flies could be used as an *in vivo* tool to search for potential therapeutic compounds against DM1 cardiac dysfunction, the effect of a known anti-DM compound was tested on the DM1 *Drosophila* cardiac dysfunction model. Several small molecules that hamper the toxic Muscleblind-CUG interaction show important anti-DM1 activity (73). Pentamidine, which has been shown to inhibit the toxic Muscleblind -CUG interaction, lessen the generation of ribonuclear foci, and release Muscleblind from the foci in treated cells, rescue partially the missplicing dysfunction of two pre-mRNAs in mice expressing CUG^exp^ *in vivo* (74) were tested in DM1 fly heart models. Pentamidine, diluted in DMSO was added to the fly food at a final concentration of 1 μM (25). The effect of Daunorubicin hydrochloride was also tested in DM1 model flies. This drug was discovered in an *in vitro* fluorescence polarization screening (70). Daunorubicin, a dsDNA and dsRNA intercalant binds competitively to the CUG repeats and inhibits MBNL1 binding. It was tested in flies under the same conditions as pentamidine. The molecular and physiological parameters were compared between the model flies treated with both compounds and with the solvent only. Flies fed with the solvent had no effect on the heart performance. In pentamidine treated flies, however, heart performance was notably improved; significant reduction in arrhythmicity and an important recovery of contractility were observed. Although affected SI and DI, representative of the systolic and diastolic dysfunction as reported in patients, were not completely rescued by pentamidine (25). Compared to pentamidine, daunorubicin treatment made a remarkable improvement in the heart performance of the model flies including SI and DI (70). Importantly, the improvement of cardiac parameters was enough to recover the median survival of the flies taking both compounds.

At the molecular level, cell and mice model experiments suggest that pentamidine and related compounds might bind the CTG.CAG repeat DNA and inhibit transcription (75). However, no significant difference in the transcript level was observed in flies taking both treatments compared to DMSO. In contrast, double FISH and immunofluorescence showed that ribonuclear inclusions were absent in cardiomyocyte nuclei and Muscleblind was distributed throughout the nucleus upon treatment. Taken together these data support that the compounds' effect was mediated by dispersing Muscleblind from sequestration rather than decreasing the expression level of toxic RNA. Indeed, the degree of recovery was different depending on the drug, e.g., pentamidine did not rescue the SI or DI but daunorubicin rescued both. Although speculative, it is tempting to suggest that differences in the extent of recovery may originate from a greater release of Muscleblind by daunorubicin than pentamidine.

The above results strongly suggest that Muscleblind sequestration contributes to heart dysfunction. To specifically address this question, Mbl isoform C (76) was overexpressed together with CUG repeats in *Drosophila* cardiomyocytes. Importantly, all the cardiac parameters including HP, AI, SI, and %FS significantly recovered in DM1 flies that overexpress Muscleblind, except for diastolic interval, that perhaps requires higher overexpression or presence of other Muscleblind protein isoforms (70).

## Concluding remarks

This review gives insight into the recent findings related to the development of *Drosophila* models to understand the pathophysiology of the DM cardiac dysfunction and search for therapeutic approaches. In short, expression of long CTG/CCTG repeats in the fly hearts reproduces conduction defects, arrhythmia and contractility defects observed in patients. Additionally, expanded repeats sequester Muscleblind, which significantly alters at least two alternative splicing events. Unlike in human patients, expanded CCTG repeat expression in fly heart generates cardiac phenotypes comparable to the alterations caused by CTG repeats suggesting that unknown modifiers in DM2 patients might be quenching the toxicity of repeats. The discovery of rbFox as modifier of DM2 muscle phenotypes (77) may shed some light on this question, as it may similarly dampen DM2 cardiac manifestations. Invertebrate models have proven that inhibition of Mbl sequestration in toxic RNA is also a valid strategy to treat cardiac defects in DM. However, further development of potential therapies is needed to provide a valid therapeutic candidate for treating DM cardiac features in humans.

## Author contributions

MC, BL, and RA have made an equal contribution to the work, and approved it for publication.

### Conflict of interest statement

The authors declare that the research was conducted in the absence of any commercial or financial relationships that could be construed as a potential conflict of interest.
